# Dual mechanism of daunorubicin-induced cell death in both sensitive and MDR-resistant HL-60 cells

**DOI:** 10.1038/sj.bjc.6690174

**Published:** 1999-03

**Authors:** M-G Côme, A Skladanowski, A K Larsen, G Laurent

**Affiliations:** CJF INSERM 9503, Institut Claudius-Régaud, 20–24 rue du Pont St-Pierre, Toulouse, 31052, France; Laboratory of Biology and Pharmacology of DNA Topoisomerases, CNRS, UMR 1772, Institut Gustave-Roussy, 39 rue Camille Desmoulin, Villejuif, 94805, France; Department of Pharmaceutical Technology and Biochemistry, Technical University of Gdansk, Narutowicza St 11/12 80–952, Gdansk, Poland; Clinical Hematology Service, CHU Purpan, place du Docteur-Baylac, Toulouse, 31059, France

**Keywords:** drug resistance, P-glycoprotein, cell death, daunorubicin, myeloid leukaemia, response prediction

## Abstract

Exposure of some acute myeloid leukaemia (AML) cells to daunorubicin leads to rapid cell death, whereas other AML cells show natural drug resistance. This has been attributed to expression of functional P-glycoprotein resulting in reduced drug accumulation. However, it has also been proposed that P-glycoprotein-expressing multidrug-resistant (MDR) cells are inherently defective for apoptosis. To distinguish between these different possibilities, we have compared the cell death process in a human AML cell line (HL-60) with a MDR subline (HL-60/Vinc) at doses that yield either similar intracellular daunorubicin concentrations or comparable cytotoxicity. Adjustment of the dose to obtain the same intracellular drug accumulation in the two cell lines did not result in equal cytotoxicity, suggesting the presence of additional resistance mechanisms in the P-glycoprotein-expressing HL-60/Vinc cells. However, at equitoxic doses, similar cell death pathways were observed. In HL-60 cells, daunorubicin induced rapid apoptosis at 0.5–1 μM and delayed mitotic cell death at 0.1 μM. These concentrations are within the clinical dose range. Similarly, HL-60/Vinc cells underwent apoptosis at 50–100 μM daunorubicin and mitotic cell death at 10 μM. These results show, for the first time, that anthracyclines can induce cell death by a dual mechanism in both sensitive and MDR cells. Our results also show that not only the cytotoxicity, but also the kinetics and mechanism of cell death, are dose dependent. Interestingly, regrowth was observed only in association with delayed cell death and the formation of enlarged, often polyploid, cells with micronucleation, suggesting that morphological criteria may be useful to evaluate treatment efficacy in patients with myeloid leukaemias. © 1999 Cancer Research Campaign


*British Journal of Cancer* (1999) **79**(7/8), 1090–1097
© 1999 Cancer Research Campaign
Article no. bjoc.1998.0174

### Dual mechanism of daunorubicin-induced cell death in both sensitive and MDR-resistant HL-60 cells

**M-G Côme[1,2], A Skladanowski[2,3], AK Larsen[2] and G Laurent[1,4]**

[1]CJF INSERM 9503, Institut Claudius-Régaud, 20–24 rue du Pont St-Pierre, 31052 Toulouse, France; [2]Laboratory of Biology and Pharmacology of DNA Topoisomerases, CNRS, UMR 1772, Institut Gustave-Roussy, PRII, 39 rue Camille Desmoulin, 94805 Villejuif, France; [3]Department of Pharmaceutical Technology and Biochemistry, Technical University of Gdansk, Narutowicza St 11/12 80–952 Gdansk, Poland; [4]Clinical Hematology Service, CHU Purpan, place du Docteur-Baylac, 31059 Toulouse, France

**Summary** Exposure of some acute myeloid leukaemia (AML) cells to daunorubicin leads to rapid cell death, whereas other AML cells show natural drug resistance. This has been attributed to expression of functional P-glycoprotein resulting in reduced drug accumulation. However, it has also been proposed that P-glycoprotein-expressing multidrug-resistant (MDR) cells are inherently defective for apoptosis. To distinguish between these different possibilities, we have compared the cell death process in a human AML cell line (HL-60) with a MDR subline (HL-60/Vinc) at doses that yield either similar intracellular daunorubicin concentrations or comparable cytotoxicity. Adjustment of the dose to obtain the same intracellular drug accumulation in the two cell lines did not result in equal cytotoxicity, suggesting the presence of additional resistance mechanisms in the P-glycoprotein-expressing HL-60/Vinc cells. However, at equitoxic doses, similar cell death pathways were observed. In HL-60 cells, daunorubicin induced rapid apoptosis at 0.5–1 μM and delayed mitotic cell death at 0.1 μM. These concentrations are within the clinical dose range. Similarly, HL-60/Vinc cells underwent apoptosis at 50–100 μM daunorubicin and mitotic cell death at 10 μM. These results show, for the first time, that anthracyclines can induce cell death by a dual mechanism in both sensitive and MDR cells. Our results also show that not only the cytotoxicity, but also the kinetics and mechanism of cell death, are dose dependent. Interestingly, regrowth was observed only in association with delayed cell death and the formation of enlarged, often polyploid, cells with micronucleation, suggesting that morphological criteria may be useful to evaluate treatment efficacy in patients with myeloid leukaemias.

**Keywords:** drug resistance; P-glycoprotein; cell death; daunorubicin; myeloid leukaemia; response prediction

Despite important progress in the understanding of the underlying molecular mechanisms, resistance to anti-neoplastic agents remains a major obstacle to curative cancer treatment. The resistance can be due to pretarget events such as drug accumulation, metabolism and intracellular drug distribution, or associated with reduced drug–target interactions. More recently, post-target events such as cell cycle progression, DNA repair and regulation of cell death have been shown to play an important role in the sensitivity of tumour cells to anti-neoplastic agents (for recent review, see Larsen and Skladanowski, 1998).

Daunorubicin is an anthracycline that is widely used in the treatment of acute myeloid leukaemia (AML). Although initially successful, resistance usually develops with time, resulting in relapse and treatment failure. Multidrug resistance (MDR) associated with overexpression of functional P-glycoprotein seems to be the most common resistance mechanism associated with relapsed AML (Baer and Bloomfield, 1991; Marie et al, 1996). The P-glycoprotein is a membrane-associated efflux pump that is able to reduce the intracellular drug accumulation of many chemically unrelated anti-tumour compounds (for review see Gottesman and Pastan, 1993).

The cytotoxic activity of daunorubicin is associated with the formation of drug-stabilized complexes between DNA and the nuclear enzyme topoisomerase II. Other biological effects include free radical formation, alkylation of DNA and interaction with components of the cell membrane (for review, see Grünicke and Hofman, 1992; Taatjes et al, 1997).

So far, little is known about the cell death process induced by daunorubicin. Current dogma is that chemotherapeutic agents induce cell death through activation of an endogenous cell death programme (programmed cell death), resulting in specific biochemical changes in the dying cells such as DNA fragmentation and cleavage of a subset of proteins usually associated with DNA repair [e.g. poly (ADP-ribose) polymerase] or maintenance of cellular structure (e.g. lamin B). This mode of cell death is accompanied by distinct morphological alterations, such as chromatin condensation and cell shrinkage, and is called apoptosis (Hickman, 1992; Sun et al, 1994). However, there is evidence to suggest that the actual situation is more complex. The topoisomerase II inhibitor etoposide can induce both apoptosis and mitotic cell death (also termed mitotic catastrophe (Lock and Ross, 1990; Lock et al, 1994)). Mitotic cell death is associated with growth arrest in the $G_2$ phase of the cell cycle followed by the formation of large, often polyploid, cells (Demarcq et al, 1994; Lock et al, 1994). In addition, etoposide exposure may lead to necrosis under ATP-depleting conditions (Eguchi et al, 1997).

Daunorubicin exposure was initially shown to induce apoptosis (Skladanowski and Konopa, 1993). More recent results show that clinical doses of daunorubicin induce apoptosis in only some acute myeloid leukaemia cells. For example, daunorubicin induces typical apoptosis in HL-60 and U937 cells but no characteristic

*Received 25 February 1998*
*Revised 11 May 1998*
*Accepted 12 June 1998*

*Correspondence to:* AK Larsen apoptotic morphological features and only very low levels of internucleosomal DNA fragmentation in the KG1 and KG1a cell lines (Quillet-Mary et al, 1996). Both KG1 and KG1a cells overexpress the *MDR-1* gene as well as functional P-glycoprotein and are naturally resistant to daunorubicin compared with HL-60 or U937 cells. It has also been reported that another anthracycline, doxorubicin, induces internucleosomal fragmentation in parental P388 leukaemia cells but not in resistant P388/ADR cells that express the P-glycoprotein (Ling et al, 1993). These results can be interpreted in different ways. It is possible that P-glycoprotein expression and an altered cell death process represent two independent resistance mechanisms, which can occur together. Alternatively, the lack of apoptosis in MDR cells might be causally linked to the transport abnormalities as a minimum intracellular concentration of anthracycline may be required to trigger apoptosis. Finally, it has been suggested that P-glycoprotein expression may somehow directly influence the apoptotic process (Ling et al, 1993; Frankfurt et al, 1994).

To distinguish between the different possibilities, we compared the daunorubicin-induced cell death process in a human AML cell line (HL-60) and a P-glycoprotein-expressing subline (HL-60/Vinc) at doses that yield either comparable intracellular daunorubicin concentrations or similar cytotoxicity. For these studies vincristine-, rather than daunorubicin-selected MDR cells were used in order to avoid possible drug-specific changes, which may occur during selection with anthracyclines, such as changes in topoisomerase II and modification of the oxidative status.

Our results show for the first time that an anthracycline can induce cell death by a dual mechanism, apoptosis or mitotic cell death, in both sensitive and MDR cells. The results also show that not only the cytotoxicity, but also the kinetics and mode of cell death are dose dependent. Interestingly, regrowth was only observed in association with delayed cell death and the formation of enlarged, often polyploid, cells with micronucleation. Therefore, morphological criteria might be useful to evaluate treatment efficacy in patients with myeloid leukaemias.

### MATERIALS AND METHODS

### Chemicals

Daunorubicin (Cerubidin®) was purchased from Laboratoire Roger Bellon (Neuilly-sur-Seine, France). Vincristine was obtained from Laboratoire Pierre Fabre (Castres, France). All other chemicals were purchased from Sigma Chemical Co. (St. Louis, MO, USA).

### Leukaemic cell lines

HL-60 cells and vincristine-selected HL-60/Vinc cells, which overexpress functional P-glycoprotein (McGrath et al, 1989), were a generous gift from Dr M Center (Kansas State University, KS, USA). HL-60/Vinc cells were maintained in the presence of 1 μM vincristine and kept in drug-free medium for at least 1 week before each experiment. HL-60 and HL-60/Vinc cells were grown in RPMI-1640 supplemented with 10% fetal calf serum (FCS), 2 mM L-glutamine and antibiotics – penicillin (100 units $ml^{-1}$) and streptomycin (100 μg $ml^{-1}$) – at 37°C in a 5% carbon dioxide/95% air atmosphere. The cells were screened routinely for *Mycoplasma* by the DNA hybridization method (Gen-Probe, San Diego, CA, USA).

### Daunorubicin accumulation

HL-60 cells ($0.5 \times 10^6$ $ml^{-1}$) were resuspended in 1 ml of RPMI with 10% FCS containing different doses of daunorubicin at 37°C. Samples were collected at the indicated times and immediately put on ice. After one wash at 4°C, cell pellets were resuspended in ice-cold phosphate-buffered saline (PBS) at $10^6$ cells $ml^{-1}$ and the daunorubicin fluorescence was determined with a Becton Dickinson FACScan flow cytometer (excitation wavelength = 488 nm; emission wavelength = 560 nm). The accumulation of daunorubicin was expressed in arbitrary units as a function of the fluorescence measured at different incubation times.

In order to confirm the cytometric measurements of intracellular daunorubicin concentrations, the drug accumulation after 1 h exposure to radiolabelled daunorubicin was also determined as previously described (Jaffrézou et al, 1991).

### Counting and viability

Cells were cultured in media containing different drug concentrations for 1 h, washed and resuspended in drug-free medium. After the specified incubation times, cells were counted with a haemocytometer, and the viability was determined by trypan blue or propidium iodide exclusion.

### Cytochemical staining

Changes in nuclear chromatin structure were evaluated by staining with 4′,6-diamidino-2-phenylindole (DAPI) as previously described (Darzynkiewicz et al, 1988). Briefly, $0.1 \times 10^6$ cells were washed once in PBS and fixed onto microscope slides by cytospin. Cells were fixed with 3% paraformaldehyde in PBS for 15 min at room temperature, washed once in PBS and then stained with PBS containing 1 μg $ml^{-1}$ DAPI for 15 min at room temperature followed by fluorescence microscopy.

### Morphological changes

Cells were exposed to daunorubicin for 1 h at 37°C, washed twice and cultured in drug-free medium. At different times, aliquots of cell suspensions were fixed onto microscope slides by cytospin and stained according to the May–Grünwald–Giemsa procedure. In order to quantify the morphologically different cell populations, 400 cells per slide were examined by light microscopy and the percentage of cells belonging to the different subpopulations was calculated and normalized with respect to $0.5 \times 10^6$ cells.

### Cell cycle analysis

All measurements were made using a Coulter EPICS Profile II flow cytometer (Coulter Electronics, Hialeah, FL, USA) equipped with an argon laser to give 488 nm light. For cell cycle distribution studies, cells were fixed in 70% ethanol, rehydrated in PBS and stained in PBS containing propidium iodide (20 μg $ml^{-1}$) and ribonuclease A (100 μg $ml^{-1}$) for 30 min at room temperature. Data from $10^4$ cells were collected and analysed by Multicycle software (Phoenix Flow Systems, San Diego, CA, USA).

### Gel electrophoresis of fragmented DNA

Following daunorubicin treatment, total cellular DNA was isolated by a previously described method (Agarwal et al, 1991) with

© Cancer Research Campaign 1999 

minor modifications. Approximately 5 × 10⁶ cells were washed in PBS, and the cell pellet was resuspended in 1 ml of a solution containing 150 mM sodium chloride, 15 mM sodium citrate, pH 7, 10 mM EDTA, 1% (w/v) sodium lauryl sarkosinate and 0.5 mg $ml^{-1}$ proteinase K. Proteolytic digestion was allowed to proceed at 50°C for 2 h. The DNA was precipitated with two volumes of absolute ethanol, resuspended in 30 µl of 10 mM Tris-HCl/1 mM EDTA buffer, pH 8, and treated with 1 mg $ml^{-1}$ RNAase for 30 min at 37°C prior to loading in a 1.8% agarose gel. Electrophoresis was carried out in 40 mM Tris-acetate/1 mM EDTA, pH 8. Gels were stained with 0.5 µg $ml^{-1}$ ethidium bromide and photographed under ultraviolet light.

### Field inversion gel electrophoresis

Field inversion gel electrophoresis was carried out as described previously (Gromova et al, 1995). After daunorubicin treatment, $2 \times 10^6$ cells were embedded in 0.75% SeaPlaque low-melting agarose (FMC Bioproducts, Rockland, ME, USA) prepared in serum-free medium. Agarose plugs were then incubated in lysis buffer [0.2 M EDTA, pH 8.0, 1% sodium dodecyl sulphate (SDS)] containing 1 mg $ml^{-1}$ proteinase K at 50°C for 36 h under gentle rotation. The plugs were washed several times in 0.2 M EDTA, pH 8.0, and stored in this solution at 4°C prior to analysis. Samples, including a 50–1000 kb lambda DNA standard (Promega Corporation, Madison, WI, USA) were analysed on a 1% agarose gel using a horizontal electrophoresis system (Gene Navigator System, Pharmacia LKB Biotechnology, Uppsala, Sweden). The pulsewave switcher was programmed to provide 15 s forward and 15 s reverse pulses for 12 h. A constant voltage of 275 V was maintained throughout the total run time in 0.5 × TBE (45 mM Tris-borate/1 mM EDTA, pH 8.6) with the temperature maintained at 4°C and continuous buffer recirculation. The gel was photographed after staining with ethidium bromide (0.5 µg $ml^{-1}$).

### TUNEL assay (terminal deoxynucleotidyl transferase assay)

Samples were prepared according to a previously published method (Gorczyca et al, 1993). Following drug treatment, cells were collected by centrifugation and fixed in 1% formaldehyde in PBS for 15 min on ice. After centrifugation, the pellet was washed in PBS, resuspended in 70% ethanol and samples were stored overnight at 4°C. After rehydration in PBS for 15 min, cells were resuspended in 50 µl of reaction buffer containing 5 units of terminal transferase, 2.5 mM cobalt chloride, 200 mM sodium cacodylate, 25 mM Tris-HCl, pH 6.6, 0.25 mg $ml^{-1}$ bovine serum albumin and 1 µg biotin-dUTP (Boehringer Mannheim, Germany) and incubated at 37°C for 30 min. Pellets were washed with rinsing buffer (PBS containing 0.1% Triton X-100, 0.5% bovine serum albumin), resuspended in 100 µl staining buffer which contained 2.5 µg $ml^{-1}$ fluorescein isothiocyanate (FITC)-avidin, 4 × SSC buffer (1 × SSC buffer: 150 mM sodium chloride, 15 mM sodium citrate, pH 7), 0.1% Triton X-100 and 5% (w/v) non-fat dry milk and incubated for 30 min at room temperature in the dark. The pellets were then washed twice with rinsing buffer, resuspended in PBS containing 5 µg $ml^{-1}$ propidium iodide and 100 µg $ml^{-1}$ RNAase A and incubated at room temperature for 30 min. The red (propidium iodide) and green (fluorescein) fluorescence were measured with EPICS Profile II flow cytometer; the data from $10^4$ cells were collected and analysed by Multigraph software.

### RESULTS

### Daunorubicin accumulation in HL-60 and HL-60/Vinc cells

We have previously shown that daunorubicin induces maximal DNA fragmentation in HL-60 cells at doses between 0.5 and 1 µM (Quillet-Mary et al, 1996). This dose range also corresponds to the peak plasma concentration after a bolus injection of daunorubicin (Speth et al, 1987). At 1 µM, the plateau in intracellular drug accumulation for HL-60 cells occurred after 60 min (Hindenburg et al, 1989). In order to determine the dose that leads to the same intracellular daunorubicin concentration in HL-60/Vinc cells as for HL-60 cells treated with 1 µM daunorubicin for 1 h, resistant cells were exposed to various doses of daunorubicin (1–100 µM) and the time course of daunorubicin accumulation was determined by flow cytometry. The results (Figure 1) show comparable drug accumulation by 1 h for HL-60 cells treated with 1 µM daunorubicin and HL-60/Vinc cells treated with 10 µM daunorubicin as expressed in peak drug accumulation. The insert (Figure 1) shows the accumulation of radiolabelled daunorubicin by 1 h and confirms the presence of comparable intracellular drug concentrations in HL-60 cells exposed to 1 µM daunorubicin and HL-60/Vinc cells exposed to 10 µM daunorubicin.

If we compare the total drug accumulation over time as expressed in AUC (total area under the intracellular drug concentration × time curve), the AUC value for HL-60/Vinc at 6 h (1 h drug exposure followed by 5 h chase in drug-free media) is between that of HL-60 treated with 1 µM and HL-60 treated with 0.5 µM daunorubicin. As no detectable drug remains in HL-60 (treated with 0.5 µM daunorubicin for 1 h) and HL-60/ Vinc (treated with 10 µM daunorubicin for 1 h) after 5 h chase in

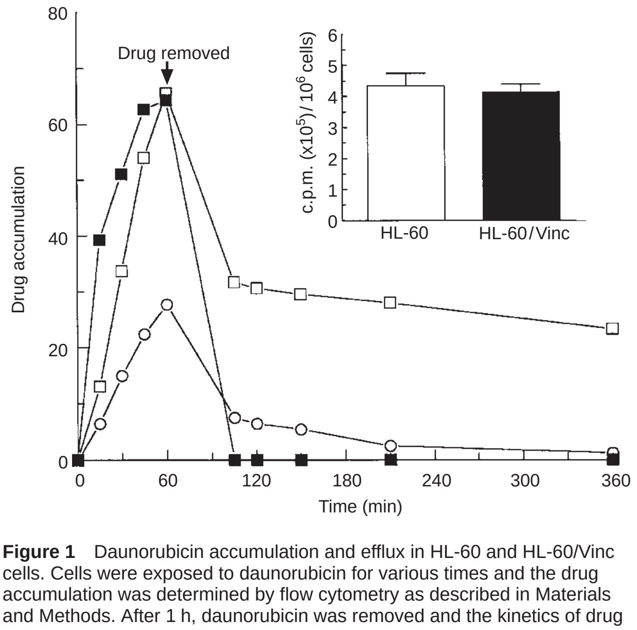

**Figure 1** Daunorubicin accumulation and efflux in HL-60 and HL-60/Vinc cells. Cells were exposed to daunorubicin for various times and the drug accumulation was determined by flow cytometry as described in Materials and Methods. After 1 h, daunorubicin was removed and the kinetics of drug efflux was followed. Daunorubicin fluorescence is expressed in arbitrary units. Data shown are typical of three independent experiments. HL-60 cells were incubated with 0.5 µM (○) or 1 µM (□) daunorubicin and HL-60/Vinc cells with 10 µM daunorubicin (■). The accumulation of [³H]daunorubicin for 1 h in HL-60 and HL-60/Vinc cells is shown in the insert. HL-60 cells were incubated with 1 µM [³H]daunorubicin and HL-60/Vinc cells with 10 µM [³H]daunorubicin. Results are the average of three independent experiments. Bars, standard deviation

© Cancer Research Campaign 1999

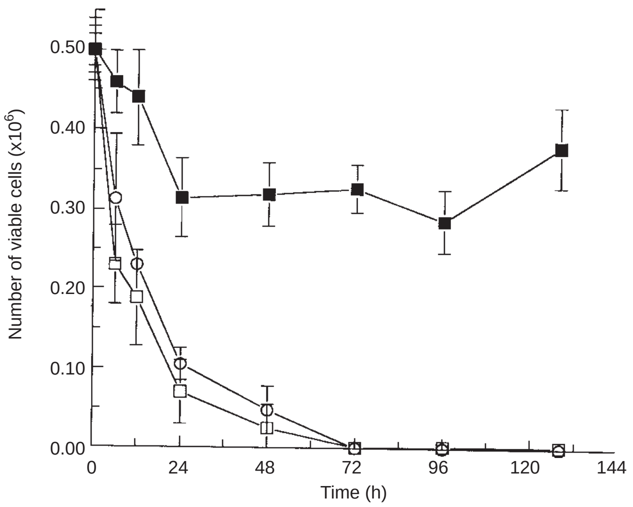

**Figure 2** The viability of HL-60 and HL-60/Vinc cells treated with daunorubicin for 1 h followed by post-incubation in drug-free medium for the indicated times, as determined by the trypan blue exclusion assay. Results are given as the mean of three independent experiments. HL-60 cells were incubated with 0.5 μM (○) or 1 μM (□) daunorubicin and HL-60/Vinc cells with 10 μM daunorubicin (■). Bars, standard deviation.

drug-free media, we can assume that these AUC values represent the total drug accumulation for the two cell lines.

### Characterization of the cell death process in daunorubicin-treated HL-60 cells

Treatment of HL-60 cells for 1 h with either 0.5 or 1 μM daunorubicin resulted in a rapid decrease in viability as determined by the trypan blue exclusion assay (Figure 2). The loss of viability was accompanied by the appearance of cells with morphological changes characteristic of apoptosis as visualized by May–Grünwald–Giemsa staining (results not shown) and by the formation of cells containing fragmented, condensed chromatin as revealed by staining with DAPI (Figure 3A). The percentage of apoptotic cells was 25%, 48% and 85% at 6, 12 and 24 h after drug treatment respectively.

Conventional agarose gel electrophoresis showed the appearance of detectable internucleosomal DNA fragmentation 4 h after daunorubicin treatment (Figure 3B), whereas field inversion gel electrophoresis showed formation of larger DNA fragments (around 50 Kbp) as early as 2 h after treatment (results not shown).

The effect of daunorubicin on cell cycle progression was also determined. Flow cytometric analysis showed that daunorubicin exposure resulted in an enrichment of cells in the early S-phase of the cell cycle as determined 6 h after drug treatment. This was accompanied by the appearance of cells in the sub-$G_1$ region, which corresponds to apoptotic cells (Figure 3C). After an additional 6 h, the number of cells in the sub-$G_1$ region had greatly increased; most remaining cells had a DNA content corresponding to early to mid-S-phase (Figure 3C). Subsequent TUNEL analysis, which identifies the fraction of cells with fragmented DNA as a function of cell cycle distribution, showed that apoptotic cells (21% by 6 h), predominantly, were recruited from $G_1$ and, to a lesser extent, from early S phase (Figure 3D).

Taken together, these results show that 1 h treatment of HL-60 cells with 1 μM daunorubicin leads to a rapid loss of viability due to apoptosis. Similar results were obtained for HL-60 cells treated with 0.5 μM daunorubicin (data not shown).

### Characterization of the cell death process in daunorubicin-treated HL-60/Vinc cells

HL-60/Vinc cells were treated with 10 μM daunorubicin for 1 h followed by incubation in drug-free medium which was renewed after 72 h. Although this dose resulted in the same intracellular drug concentration as for HL-60 cells treated with 0.5 to 1 μM daunorubicin (Figure 1), the cellular effects were very different. The cell viability decreased by up to 50% during the first 24 h, after which time the number of viable cells remained almost constant for the next 72 h (Figure 2). After 96 h, the cells started to grow again and had recovered normal proliferation rate by 144 h (data not shown). Light microscopy studies showed that different morphological changes occurred during the post-treatment period. Three major populations could be identified: cells with unchanged morphology, enlarged/polyploid cells and apoptotic cells (Figure 4A). As shown in Figure 5, enlarged cells appeared rapidly and represented about 60% of the total cell population 12 h after drug treatment. This was followed by the progressive emergence of polyploid cells that often had fragmented nuclei. Thereafter, the number of large cells decreased and only few such cells could be detected after 120 h. A small fraction of apoptotic cells was observed throughout the post-treatment period, the percentage of which increased to reach 20% of the total cell population 96 h after drug treatment. Finally, the fraction of cells with normal morphology was strongly reduced during the first 96 h and then started to increase, constituting the majority of cells by 120 h. Interestingly, surviving cells exhibited P-glycoprotein expression levels, drug sensitivity and morphological features similar to those of untreated HL-60/Vinc cells, suggesting that the surviving cells did not represent a subpopulation with intrinsic higher daunorubicin resistance (results not shown).

DNA analysis by both conventional and pulse-field gel electrophoresis revealed no DNA fragmentation until 48 h after treatment, when both large DNA fragments (not shown) and DNA ladders (Figure 4B) appeared.

Flow cytometric analysis of daunorubicin-treated cells revealed a marked growth arrest of cells in the $G_2$/M phase of the cell cycle 24 h after drug treatment (Figure 4C); thereafter, cells with 8N DNA content were detected (Figure 4C). In parallel, cells appeared in the sub-$G_1$-region, which corresponds to apoptotic cells. TUNEL analysis confirmed that DNA fragmentation was a slow event, which first occurred 24 h after drug treatment, and then slowly increased to include 35% of the cells after 132 h. The DNA fragmentation preferentially occurred in late S-phase, whereas no apparent fragmentation took place in cells with DNA ploidy higher than 4N (Figure 4D). To determine what happened with both normal and enlarged cells, cell sorting experiments were carried out 72 h after drug treatment based on side and forward scatter, separating cells with 2N and cells with >4N DNA content. Subsequent incubation of the two cell populations revealed that the large, mostly polyploid cells eventually died out whereas the cells with 2N DNA content started to grow again (result not shown).

These results show that treatment of HL-60/Vinc cells with 10 μM daunorubicin induced a $G_2$/M arrest, which was followed by either polyploidy or delayed internucleosomal DNA fragmentation

© Cancer Research Campaign 1999

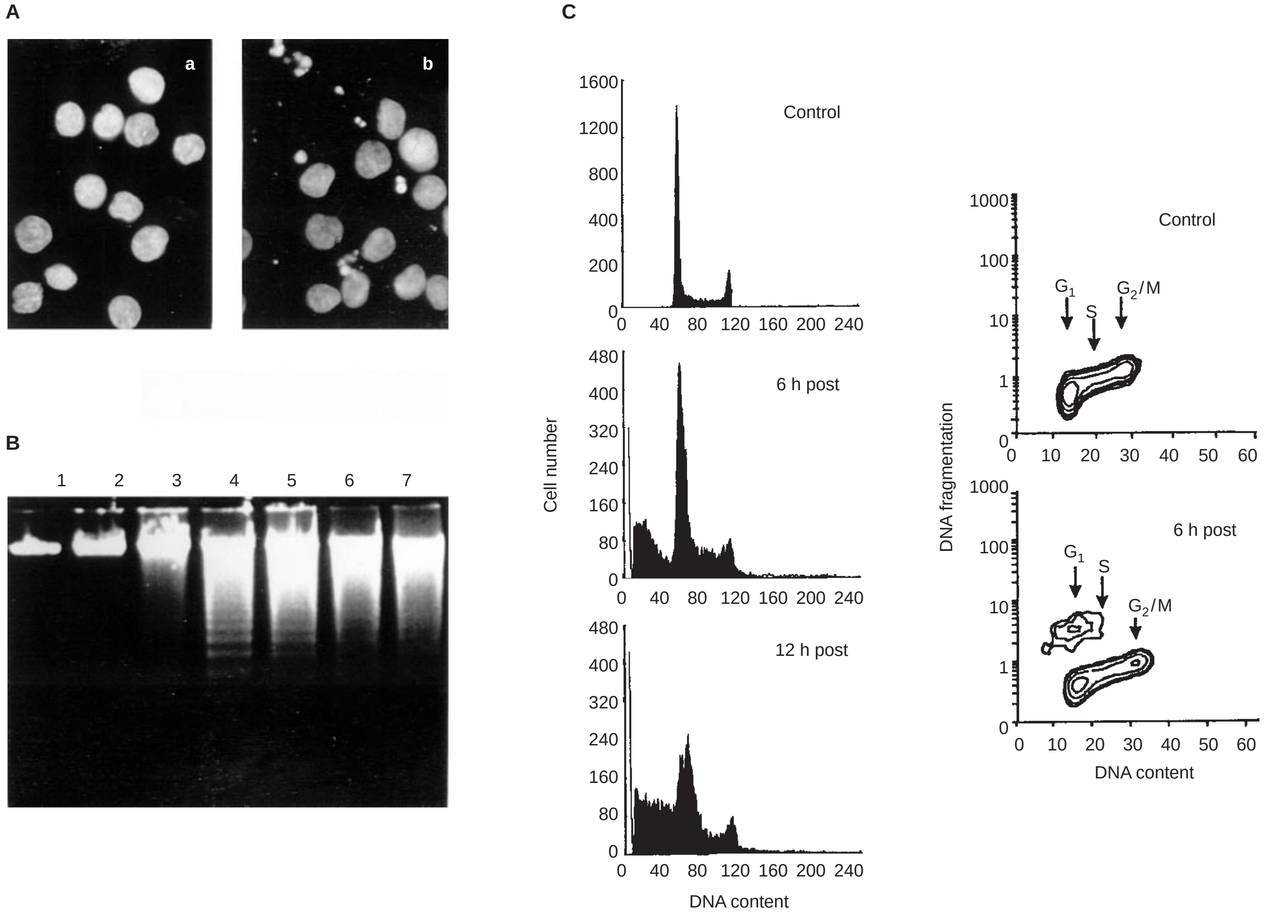

**Figure 3** Nuclear morphology, DNA fragmentation and cell cycle distribution of HL-60 cells exposed to 1 μM daunorubicin for 1 h, followed by post-incubation in drug-free medium for the indicated times. Data shown are typical of three independent experiments. (**A**) Morphological alterations of chromatin were evaluated by DAPI staining and viewed at 400 × magnification: (a) untreated cells, (b) daunorubicin-treated cells after 6 h post-incubation. Note the presence of numerous apoptotic cells, e.g. top left. (**B**) Formation of oligonucleosomal DNA fragments. DNA was isolated at various times and analysed by agarose gel electrophoresis. Lane 1, control (untreated cells); lanes 2–7, cells after 2, 4, 6, 12, 24 and 48 h post-incubation. (**C**) Cell cycle distribution (**D**) DNA fragmentation of HL-60 cells after 6 h post-incubation in drug-free medium as determined by the TUNEL assay that occurred during the next cell cycle. This sequence of events was clearly very different from what was observed for HL-60 cells treated with 0.5 to 1 μM daunorubicin although the overall intracellular drug concentration was comparable between the two cell lines. One explanation could be that HL-60/Vinc cells have additional resistance mechanisms such as an altered intracellular distribution or an altered topoisomerase II, which would lead to decreased drug target interaction. Alternatively, it could be a direct result of differences in the cell death programme between the two cell lines.

### Dose effect of daunorubicin on the cell death process in HL-60 and HL-60/Vinc cells

To establish if the cell death pathway was modified in the resistant cells, HL-60 and HL-60/Vinc cells were treated with isotoxic doses of daunorubicin. The previous treatment with 1 μM daunorubicin for HL-60 cells or 10 μM for HL-60/Vinc cells resulted in approximately 90% ($IC_{90}$) and 50% ($IC_{50}$) loss of viability, respectively, as determined by trypan blue exclusion by 24 h (Figure 2).

Treatment of HL-60 cells with 0.1 μM daunorubicin, which corresponds to the $IC_{50}$ dose for this cell line, resulted in a $G_2$/M block, followed by the occurrence of polyploid cells, delayed DNA fragmentation (Figure 6A) and a transient decrease in cell viability. These findings are similar to what was observed for HL-60/Vinc cells treated with an isotoxic dose of daunorubicin (10 μM).

Alternatively, HL-60/Vinc cells were treated with 100 μM daunorubicin, which corresponds to the $IC_{90}$ value for this cell line. This resulted in rapid apoptosis, with no arrest in $G_2$/M but a rapid accumulation of cells in $G_1$ and early S followed by internucleosomal DNA fragmentation (Figure 6B). These results are similar to what was observed for HL-60 cells treated with an isotoxic dose of daunorubicin (0.5–1 μM).

Altogether, these studies show that daunorubicin may induce rapid apoptosis or delayed cell death in both sensitive and MDR-resistant cells depending on dose. No differences in the cell death processes were observed between the two cell lines at isotoxic concentrations. We conclude that neither modifications of the cell cycle regulation nor of the cell death process had occurred in the multidrug-resistant HL-60/Vinc cells as determined by the cellular response to daunorubicin.

*© Cancer Research Campaign 1999*

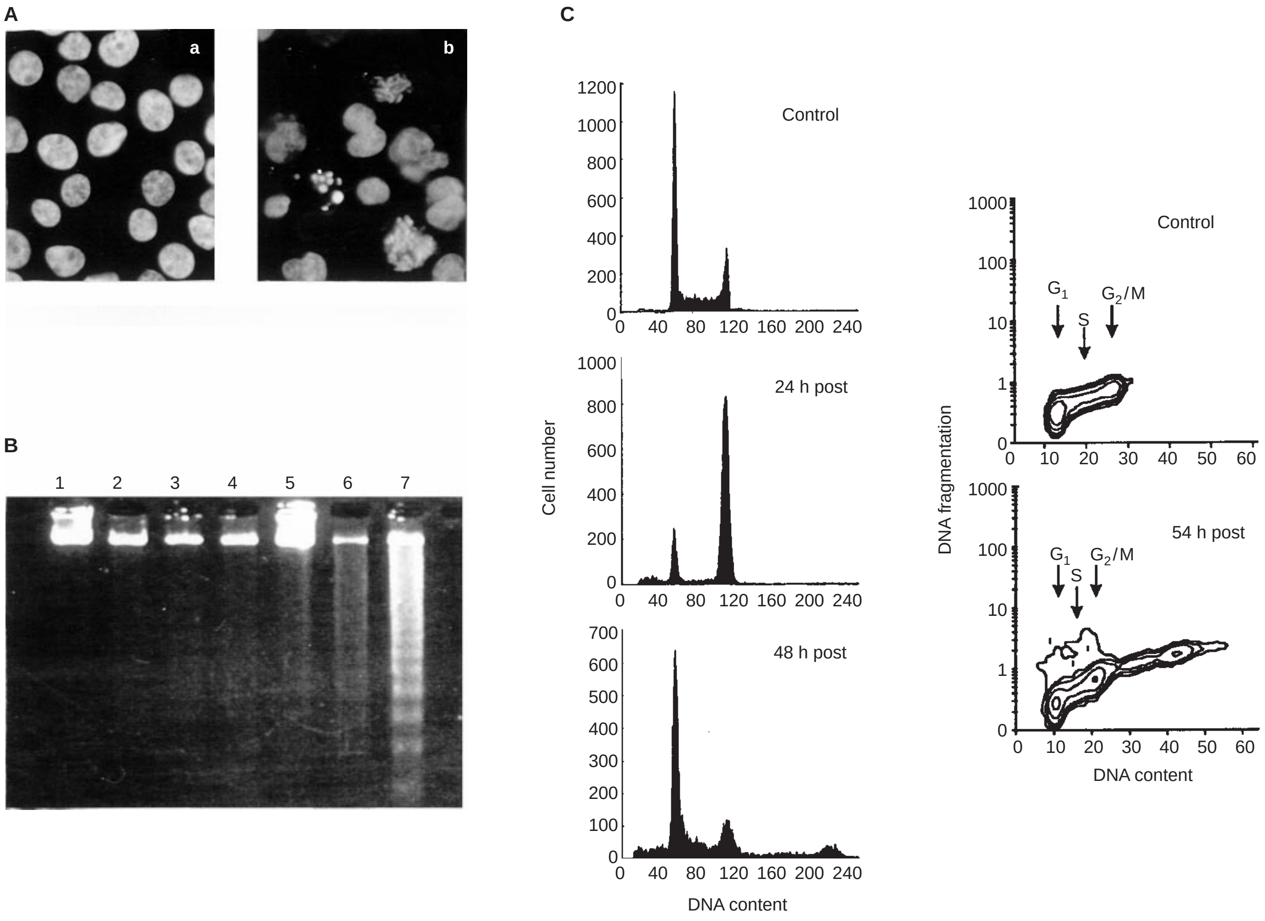

**Figure 4** Nuclear morphology, DNA fragmentation and cell cycle distribution of HL-60/Vinc cells treated with 10 μM daunorubicin for 1 h, followed by incubation in drug-free medium for the indicated times. Data shown are typical of three independent experiments. (**A**) Morphological alterations of chromatin were evaluated by DAPI staining and viewed at 400 × magnification: (a) untreated control cells, (b) daunorubicin-treated cells after 48 h post-incubation. Note the presence of apoptotic cells (middle left), normal mitotic figures (top) and enlarged cells with multiple nuclei (middle right) of which one is undergoing mitosis (bottom right). (**B**) Formation of oligonucleosomal DNA fragments. DNA was isolated at various times and analysed by agarose gel electrophoresis. Lane 1, control (untreated cells); lanes 2–7, cells after 2, 4, 6, 12, 24, 48 h post-incubation. (**C**) Cell cycle distribution. (**D**) DNA fragmentation of HL-60/Vinc cells after 54 h post-incubation in drug-free medium, as determined by the TUNEL assay

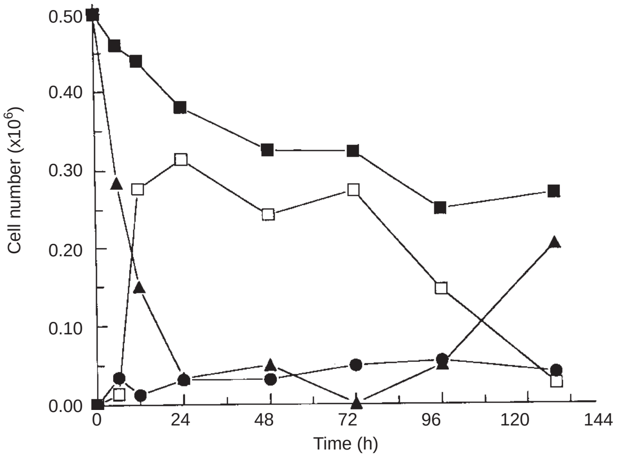

**Figure 5** Morphological changes of HL-60/Vinc cells exposed to 10 μM daunorubicin for 1 h, followed by incubation in drug-free medium. The viability of HL-60/Vinc cells was determined by trypan blue exclusion at the indicated times. In parallel, cells were stained according to the May–Grünwald–Giemsa procedure. For each time point 400 cells were examined, the percentage of cells belonging to the different subpopulations was determined and normalized with respect to the total number of viable cells. Data shown are typical of three independent experiments. Cells with unchanged morphology (▲), enlarged cells (□), apoptotic cells (●) and total cell number (■)

### DISCUSSION

Exposure of some AML cells to daunorubicin leads to rapid apoptotic cell death, whereas other AML cells show natural resistance, which has been attributed to the expression of functional P-glycoprotein resulting in decreased drug accumulation (Quillet-Mary et al, 1996). However, it has also been proposed that P-glycoprotein-expressing MDR cells are inherently defective for apoptosis (Ling et al, 1993; Frankfurt et al, 1994). The aim of the present study was to compare the cell death pathways in a human AML cell line (HL-60) with its MDR counterpart (HL-60/Vinc) at doses that yield either comparable intracellular daunorubicin concentrations or comparable cytotoxicity. We showed that a clinically relevant exposure of HL-60 cells to daunorubicin (0.5–1 μM for 1 h) leads to rapid apoptosis. In contrast, when HL-60 cells were treated with 0.1 μM daunorubicin for 1 h, a different type of cell death was observed, which has the features of mitotic cell death. Mitotic cell death is a slow process that requires several cell cycles and is associated with the formation of enlarged, often polyploid and multinucleated, cells. Most of the cells eventually die by mitotic failure ('mitotic cell death'), whereas a smaller fraction dies by delayed apoptosis.

© Cancer Research Campaign 1999

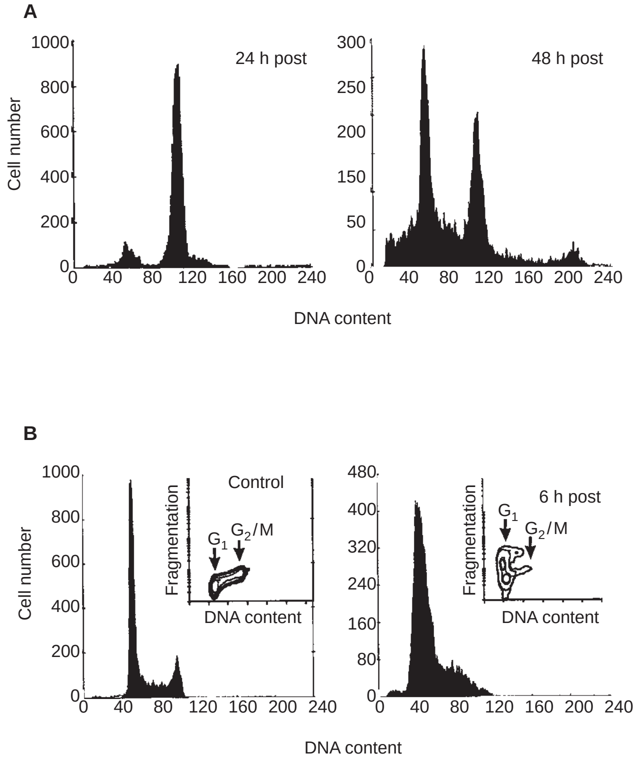

**Figure 6** Cell cycle distribution of (**A**) HL-60 cells exposed to 0.1 μM daunorubicin for 1 h followed by post-incubation in drug-free medium for the indicated times. (**B**) HL-60/Vinc cells exposed to 100 μM daunorubicin for 1 h followed by post-incubation in drug-free medium for the indicated times. Insert, DNA fragmentation of HL-60/Vinc cells as determined by the TdT assay

The mechanism of cell death was also dose dependent in HL-60/Vinc cells, although much higher drug concentrations were required to induce the two processes. Mitotic cell death was observed after 1-h treatment with 10 μM daunorubicin, while apoptosis occurred at 50–100 μM daunorubicin. Therefore, our results do not support the notion that P-glycoprotein expressing MDR cells are inherently defective for apoptosis, as has previously been suggested (Ling et al, 1993; Frankfurt et al, 1994).

Although no intrinsic changes in the regulation of cell cycle progression or cell death pathways occurred in HL-60/Vinc cells, our results show that the resistance of these cells is at least bifactorial because HL-60/Vinc cells remain refractory to daunorubicin even at intracellular drug concentrations that are sufficient to kill sensitive cells. These results were unexpected as it has been reported that expression of mdr1 antisense oligodeoxynucleotides in HL-60/Vinc cells totally restores their sensitivity towards vincristine (Cucco and Calabretta, 1996). No difference between HL-60 and HL-60/Vinc cells was found with respect to both multidrug-related protein (MRP) and lung-related protein (LRP) expression (M.-G. Côme, A. Skladanowski and A.K. Larsen, unpublished results). It is possible that the resistant cells have an altered intracellular drug distribution, as sequestration of anthracyclines into cytoplasmic vesicles is well documented for many cell lines with acquired drug resistance (Keizer et al, 1989; Gervasoni et al, 1991; Meschini et al, 1994; Seidel et al, 1995). An additional possibility is that the nuclear target for daunorubicin, DNA topoisomerase II, could be altered in the HL-60/Vinc cells.

To the best of our knowledge, this is the first report describing a dual mode of cell death induced by an anthracycline. A similar situation has previously been reported for other DNA-damaging agents, including etoposide, X-ray irradiation, bleomycin and cisplatin (Lock and Ross, 1990; Chang and Little, 1992; Rodilla, 1993; Tounekti et al, 1993; Thompson, 1995). The dual mode of cell death might have impact on the clinical efficiency of these compounds because the two cell death programmes appear to be under separate genetic control. This was most clearly shown by recent experiments when the overexpression of Bcl-2 in human epithelial tumour cells inhibited etoposide-induced apoptosis, but had no effect on the formation of polyploid, multinucleated cells characteristic of mitotic cell death. As a consequence, Bcl-2 enhanced short-term viability but had no effect on long-term clonogenic survival (Lock and Stribinskiene, 1996).

It is well established that the apoptotic response is affected by multiple stimuli, including the expression of oncogenes and tumour-suppressor genes and the presence of external survival factors (Thompson, 1995). In contrast, the mechanism of mitotic cell death is less well understood. Anthracycline treatment leads to cell cycle arrest in either the $G_1$ or $G_2$ phase of the cell cycle depending on dose and cell type (Tobey, 1972; Bhunyan and Groppi, 1989). In our system, no $G_1$ arrest was observed within a large range of concentrations, which is probably related to the absence of the p53 gene in HL-60 cells (Sugimoto et al, 1992). Cell cycle arrest in the $G_2$ phase is believed to protect the cell by providing time to repair DNA lesions prior to mitosis and the initiation of a new cell cycle. In principle, the $G_2$ arrest must consist of at least three components: a sensor that monitors the integrity of the genome, a signal that this sensor generates and a response element in the cell cycle engine that causes it to arrest or delay before the $G_2$ to M transition. In budding yeast, several genes including *RAD9*, *RAD17*, *RAD24* and *MEC3* have been identified in which gene products participate in the feedback control that detects damaged DNA. Cells that contain recessive mutations in these genes do not arrest in $G_2$ subsequent to DNA damage and suffer lethal chromosome damage during mitosis (Weinert and Hartwell, 1988; Weinert, 1992). The response element that causes $G_2$ arrest following treatment with daunorubicin is not known. However, it has been reported that treatment with other topoisomerase inhibitors such as doxorubicin (Ling et al, 1996), etoposide (Lock and Ross, 1990) and camptothecin (Tsao et al, 1992) prevents the activation of $p34^{cdc2}$ kinase, which controls the entry into mitosis. Addition of caffeine to etoposide-treated cells leads to activation of $p34^{cdc2}$, release of the cells from $G_2$ arrest and subsequent increase in the incidence of mitotic death (Lock et al, 1994). Taken together, these results suggest that mitotic cell death may arise from a failure of coupling the $G_2$/M transition to completion of DNA repair.

Our results have several important practical implications. The doses that induce either rapid apoptosis or delayed mitotic cell death in HL-60 cells are both clinically relevant as a bolus administration of daunorubicin to leukaemia patients results in a mean peak plasma concentration of about 0.5 μM (Speth et al, 1987). Therefore, a relatively modest reduction in the in vivo daunorubicin peak plasma concentration due to a reduced dose, slow-down of drug administration or altered pharmacokinetics could have a profound effect on the efficiency of the treatment. Our results may also be relevant for response prediction. In both sensitive and resistant cells, the two different cell death pathways resulted in a different outcome as regrowth was observed only in association

 © Cancer Research Campaign 1999

with mitotic cell death, which is characterized by the formation of enlarged, often polyploid, cells with micronucleation. Therefore, morphological criteria might be a useful way to estimate treatment efficacy in patients with myeloid leukaemia.

### ACKNOWLEDGEMENTS

We are grateful to Arlette Verwish for help with flow cytometry measurements. This work was supported by grants from la Fédération des Centres de Lutte Contre le Cancer (GL), Association pour la Recherche sur le Cancer (AKL, GL), Ligue Nationale contre le Cancer (AKL), FEGEFLUC (AKL) and INSERM (GL). AS is a Fellow of la Fondation pour la Recherche Médicale.

### ABBREVIATIONS

AML, acute myeloid leukaemia; MDR, multidrug-resistant; FCS, fetal calf serum; PBS, phosphate-buffered saline; DAPI, 4′,6-diamidino-2-phenylindole; SDS, sodium dodecyl sulphate; EDTA, ethylenediaminetetraacetic acid; TdT, terminal deoxynucleotidyl transferase; MRP, multidrug-related protein; LRP, lung-related protein.

### REFERENCES

Agarwal ML, Clay ME, Harvey EJ, Evans HH, Antunez AR and Oleinick NL (1991) Photodynamic therapy induces rapid cell death by apoptosis in L5178Y mouse lymphoma cells. *Cancer Res* **51**: 5993–5996

Baer MR and Bloomfield CD (1991) Multidrug resistance in acute myeloid leukemia. *J Natl Cancer Inst* **83**: 663–665

Bhuyan BK and Groppi VE (1989) Cell cycle specific inhibitors. *Pharmacol Ther* **42**: 307–348

Chang WP and Little JB (1992) Delayed reproductive death as a dominant phenotype in cell clones surviving X-irradiation. *Carcinogenesis* **13**: 923–928

Cucco C and Calabretta B (1996) In vitro and in vivo reversal of multidrug resistance in a human leukemia-resistant cell line by mdr1 antisense oligodeoxynucleotides. *Cancer Res* **56**: 4332–4337

Darzynkiewicz Z, Carter SP, Mikulski SM, Ardelt WJ and Shogen K (1988) Cytostatic and cytotoxic effects of Pannon (P-30 Protein), a novel anticancer agent. *Cell Tissue Kinet* **21**: 169–182

Demarcq C, Bunch RT, Creswell D and Eastman A (1994) The role of cell cycle progression in cisplatin-induced apoptosis in Chinese hamster ovary cells. *Cell Growth Differ* **5**: 983–993

Eguchi Y, Shimizu S and Tsujimoto Y (1997) Intracellular ATP levels determine cell death fate by apoptosis or necrosis. *Cancer Res* **57**: 1835–1840

Frankfurt OS, Seckinger D and Sugarbaker EV (1994) Pleiotropic drug resistance and survival advantage in leukemic cells with diminished apoptotic response. *Int J Cancer* **59**: 217–224

Gervasoni JE Jr, Fields SZ, Krishna S, Baker MA, Rosado M, Thuraisamy K, Hindenburg AA and Taub RN (1991) Subcellular distribution of daunorubicin in P-glycoprotein-positive and -negative drug-resistant cell lines using laser-assisted confocal microscopy. *Cancer Res* **51**: 4955–4963

Gorczyca W, Gong J and Darzynkiewicz Z (1993) Detection of DNA strand breaks in individual apoptotic cells by the in situ terminal deoxynucleotidyl transferase and nick translation assays. *Cancer Res* **53**: 1945–1951

Gottesman MM and Pastan I (1993) Biochemistry of multidrug resistance mediated by the multidrug transporter. *Annu Rev Biochem* **62**: 385–427

Gromova II, Thomsen B and Razin SV (1995) Different topoisomerase II antitumor drugs direct similar specific long-range fragmentation of an amplified c-MYC gene locus in living cells and in high-salt-extracted nuclei. *Proc Natl Acad Sci USA* **92**: 102–106

Grunicke H and Hofmann J (1992) Cytotoxic and cytostatic effects of antitumor agents induced at the plasma membrane level. *Pharmacol Ther* **55**: 1–30

Hickman JA (1992) Apoptosis induced by anticancer drugs. *Cancer Metastasis Rev* **11**: 121–139

Hindenburg AA, Gervasoni JE Jr, Krishna S, Stewart VJ, Rosado M, Lutzky J, Bhalla K, Baker MA and Taub RN (1989) Intracellular distribution and pharmacokinetics of daunorubicin in anthracycline-sensitive and -resistant HL-60 cells. *Cancer Res* **49**: 4607–4614

Jaffrezou JP, Herbert JM, Levade T, Gau MN, Chatelain P and Laurent G (1991) Reversal of multidrug resistance by calcium channel blocker SR33557 without photoaffinity labeling of P-glycoprotein. *J Biol Chem* **266**: 19858–19864

Keizer HG, Schuurhuis GJ, Broxterman HJ, Lankelma J, Schoonen WG, Van Rijn J, Pinedo HM and Joenje H (1989) Correlation of multidrug resistance with decreased drug accumulation, altered subcellular drug distribution, and increased P-glycoprotein expression in cultured SW-1573 human lung tumor cells. *Cancer Res* **49**: 2988–2993

Larsen AK and Skladanowski A (1998) Cellular resistance to topoisomerase targeted drugs: from drug uptake to cell death. *Biochim Biophys Acta (Gen Struct Exp)* **1400**: 257–274

Ling YH, Priebe W and Perez-Soler R (1993) Apoptosis induced by anthracycline antibiotics in P388 parent and multidrug-resistant cells. *Cancer Res* **53**: 1845–1852

Ling YH, El-Naggar AK, Priebe W and Perez-Soler R (1996) Cell cycle-dependent cytotoxicity, G2/M phase arrest, and disruption of p34cdc2/cyclin B1 activity induced by doxorubicin in synchronized P388 cells. *Mol Pharmacol* **49**: 832–841

Lock RB and Ross WE (1990) Possible role for p34cdc2 kinase in etoposide-induced cell death of Chinese hamster ovary cells. *Cancer Res* **50**: 3767–3771

Lock RB, Galperina OV, Feldhoff RC and Rhodes LJ (1994) Concentration-dependent differences in the mechanisms by which caffeine potentiates etoposide cytotoxicity in HeLa cells. *Cancer Res* **54**: 4933–4939

Lock RB and Stribinskiene L (1996) Dual modes of death induced by etoposide in human epithelial tumor cells allow Bcl-2 to inhibit apoptosis without affecting clonogenic survival. *Cancer Res* **56**: 4006–4012

Marie JP, Zhou DC, Gurbuxani S, Legrand O and Zittoun R (1996) MDR1/P-glycoprotein in haematological neoplasms. *Eur J Cancer* **32A**: 1034–1038

McGrath T, Latoud C, Arnold ST, Safa AR, Felsted RL and Center MS (1989) Mechanisms of multidrug resistance in HL60 cells. Analysis of resistance associated membrane proteins and levels of mdr gene expression. *Biochem Pharmacol* **38**: 3611–3619

Meschini S, Molinari A, Calcabrini A, Citro G and Arancia G (1994) Intracellular localization of the antitumour drug adriamycin in living cultured cells: a confocal microscopy study. *J Microsc* **176**: 204–210

Quillet-Mary A, Mansat V, Duchayne E, Côme MG, Allouche M, Bailly JD, Bordier C and Laurent G (1996) Daunorubicin-induced internucleosomal DNA fragmentation in acute myeloid cell lines. *Leukemia* **10**: 417–425

Rodilla V (1993) Origin and evolution of binucleated cells and binucleated cells with micronuclei in cisplatin-treated CHO cultures. *Mutat Res* **300**: 281–291

Seidel A, Hasmann M, Loser R, Bunge A, Schaefer B, Herzig I, Steidtmann K and Dietel M (1995) Intracellular localization, vesicular accumulation and kinetics of daunorubicin in sensitive and multidrug-resistant gastric carcinoma EPG85–257 cells. *Virchow's Arch* **426**: 249–256

Skladanowski A and Konopa J (1993) Adriamycin and daunomycin induce programmed cell death (apoptosis) in tumour cells. *Biochem Pharmacol* **46**: 375–382

Speth PA, Linssen PC, Boezeman JB, Wessels HM and Haanen C (1987) Leukemic cell and plasma daunomycin concentrations after bolus injection and 72 h infusion. *Cancer Chemother Pharmacol* **20**: 311–315

Sugimoto K, Toyoshima H, Sakai R, Miyagawa K, Hagiwara K, Ishikawa F, Takaku F, Yazaki Y and Hirai H (1992) Frequent mutations in the p53 gene in human myeloid leukemia cell lines. *Blood* **79**: 2378–2383

Sun XM, Snowden RT, Dinsdale D, Ormerod MG and Cohen GM (1994) Changes in nuclear chromatin precede internucleosomal DNA cleavage in the induction of apoptosis by etoposide. *Biochem Pharmacol* **47**: 187–195

Taatjes DJ, Gaudiano G, Resing K and Koch TH (1997) Redox pathway leading to the alkylation of DNA by the anthracycline, antitumor drugs adriamycin and daunomycin. *J Med Chem* **40**: 1276–1286

Thompson CB (1995) Apoptosis in the pathogenesis and treatment of disease. *Science* **267**: 1456–1462

Tobey RA (1972) Effects of cytosine arabinoside, daunomycin, mithramycin, azacytidine, adriamycin, and camptothecin on mammalian cell cycle traverse. *Cancer Res* **32**: 2720–2725

Tounekti O, Pron G, Belehradek J Jr and Mir LM (1993) Bleomycin, an apoptosis-mimetic drug that induces two types of cell death depending on the number of molecules internalized. *Cancer Res* **53**: 5462–5469

Tsao YP, D'Arpa P and Liu LF (1992) The involvement of active DNA synthesis in camptothecin-induced G2 arrest: altered regulation of p34cdc2/cyclin B. *Cancer Res* **52**: 1823–1829

Weinert TA (1992) Dual cell cycle checkpoints sensitive to chromosome replication and DNA damage in the budding yeast *Saccharomyces cerevisiae*. *Radiat Res* **132**: 141–143

Weinert TA and Hartwell LH (1988) The RAD9 gene controls the cell cycle response to DNA damage in *Saccharomyces cerevisiae*. *Science* **241**: 317–322

© Cancer Research Campaign 1999
